# Supplementary material for: Systems biology-enabled targeting of NF-κΒ and BCL2 overcomes microenvironment-mediated BH3-mimetic resistance in DLBCL
Source: Cell Death Dis. 2025 Aug 16;16(1):620. doi: 10.1038/s41419-025-07942-0 (PMC12357900; doi:10.1038/s41419-025-07942-0)
Supplement: Supplementary file 1 — Supplemental Material [file 41419_2025_7942_MOESM1_ESM.pdf]

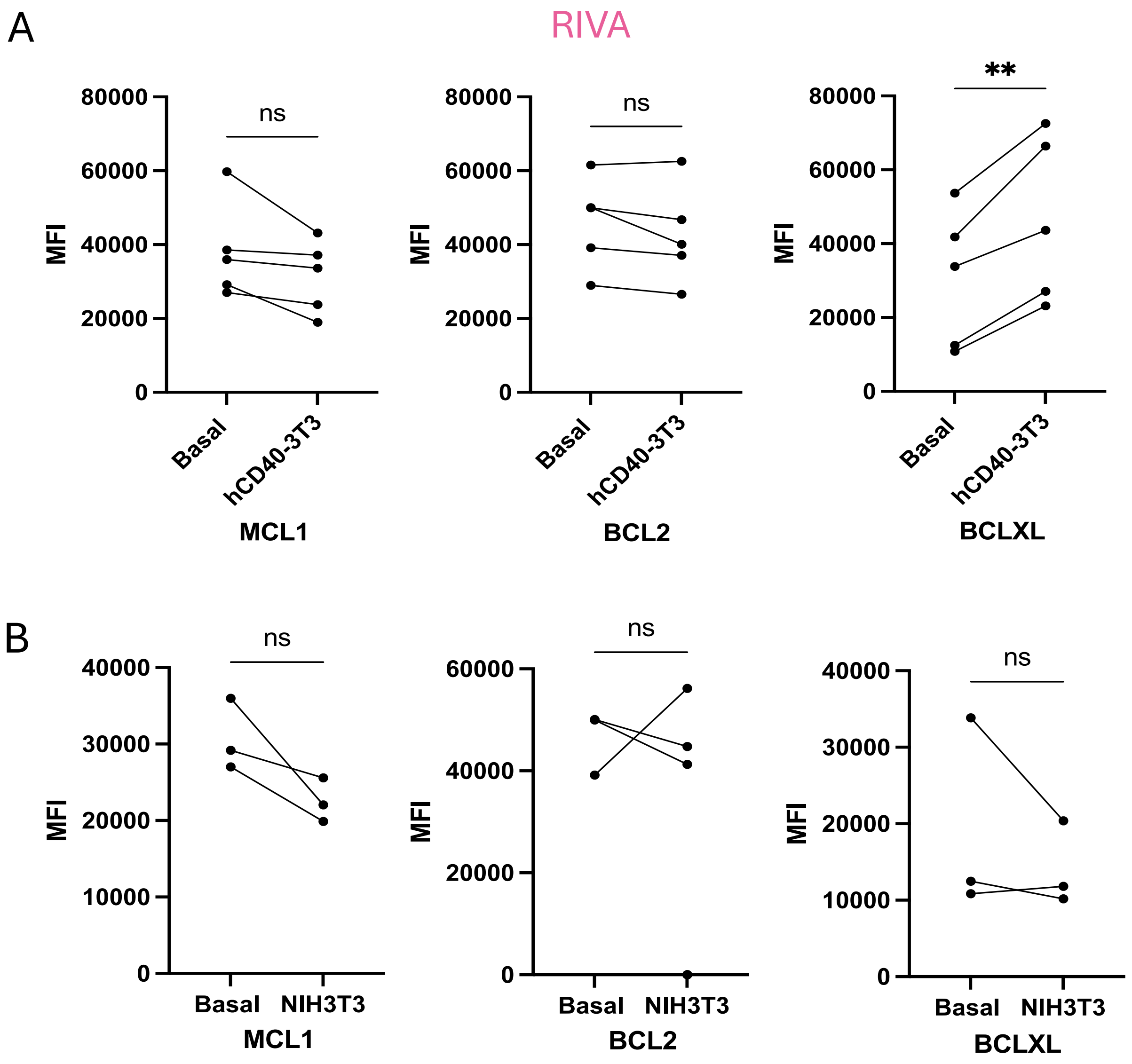

Supplemental Figure 1. Higher anti-apoptotic levels are seen in DLBCL cells when are co-cultured with CD40L transfected fibroblasts. (A) RIVA cells were incubated for 24 hours with or without the presence of CD40L transfected fibroblasts. After the stimulation, the DLBCL cells were harvested and stained for Mcl-1, Bcl-2 and Bcl-xL, and then assessed by using flow cytometry. Statistical analysis was performed on five independent experiments' Mean Fluorescence Intensity (MFI) values, between unstimulated and CD40L stimulated RIVA cells, (***P*<0.01, paired T-test). (B) RIVA cells were incubated for 24 hours with or without the presence of fibroblasts. After the stimulation, the DLBCL cells were harvested and stained for Mcl-1, Bcl-2 and Bcl-xL, and then assessed by using flow cytometry. Statistical analysis was performed on three independent experiments Mean Fluorescence Intensity (MFI) values, between the monocultured and co-cultured RIVA cells, (paired T-test).

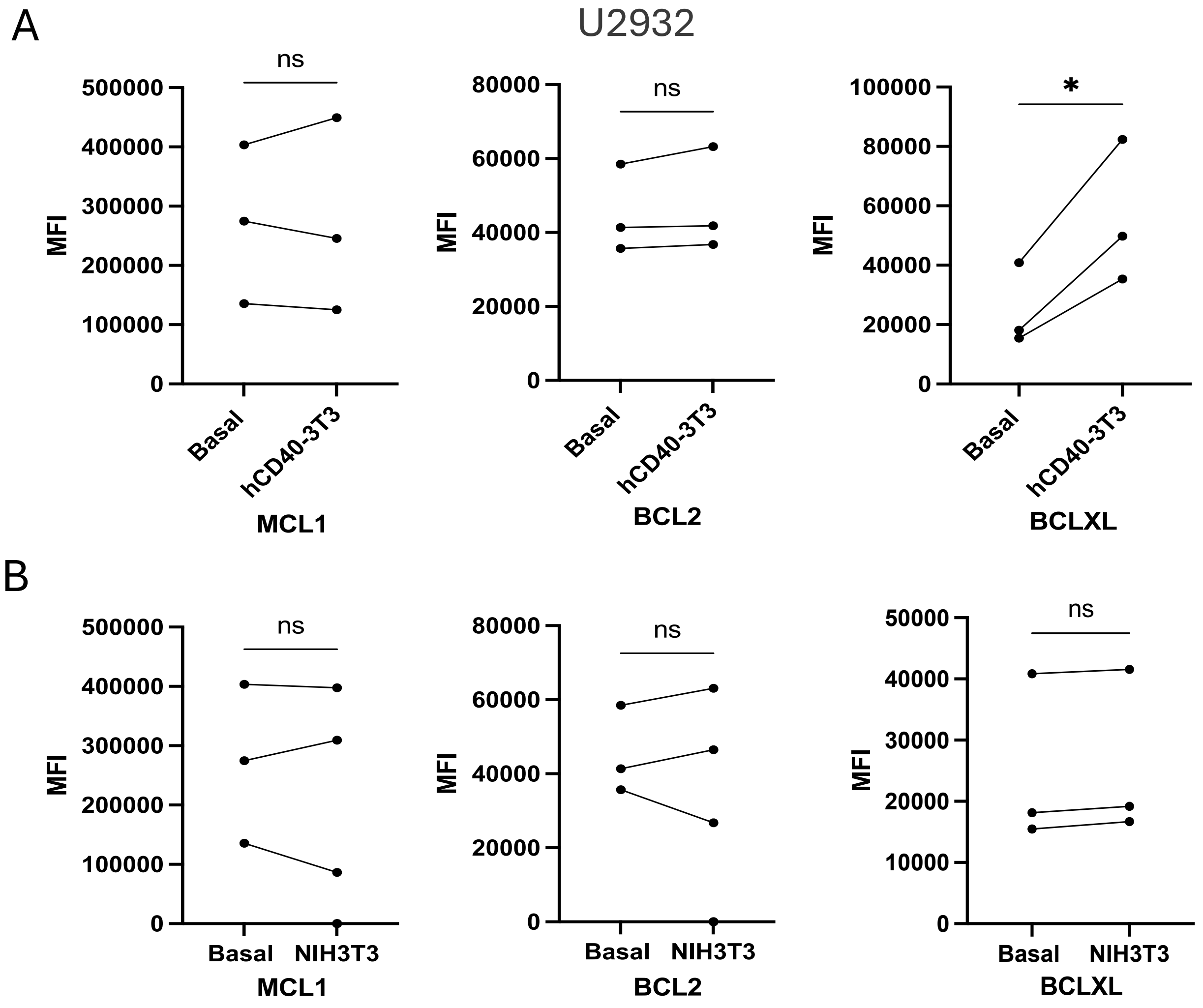

Supplemental Figure 2. Higher anti-apoptotic levels are seen in DLBCL cells when are co-cultured with CD40L transfected fibroblasts. (A) U2932 cells were incubated for 24 hours with or without the presence of CD40L transfected fibroblasts. After the stimulation, the DLBCL cells were harvested and stained for Mcl-1, Bcl-2 and Bcl-xL, and then assessed by using flow cytometry. Statistical analysis was performed on three independent experiments' Mean Fluorescence Intensity (MFI) values, between unstimulated and CD40L stimulated U2932 cells, (*P<0.05, **P<0.01 paired T-test). (B) U2932 cells were incubated for 24 hours with or without the presence of fibroblasts. After the stimulation, the DLBCL cells were harvested and stained for Mcl-1, Bcl-2 and Bcl-xL, and then assessed by using flow cytometry. Statistical analysis was performed on three independent experiments Mean Fluorescence Intensity (MFI) values, between the monocultured and co-cultured U2932 cells, (paired T-test).

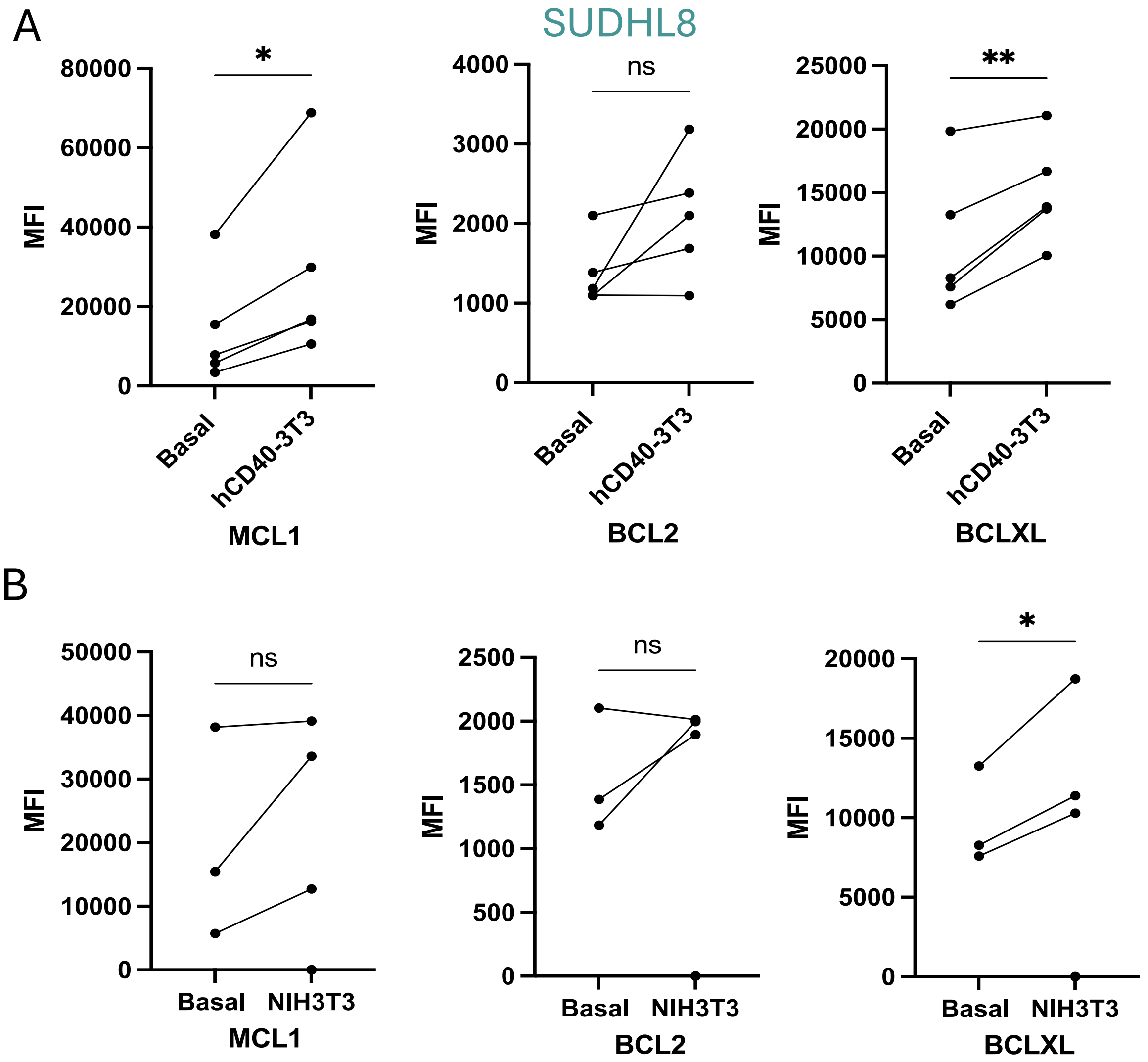

Supplemental Figure 3. Higher anti-apoptotic levels are seen in DLBCL cells when are co-cultured with CD40L transfected fibroblasts. (A) SUDHL8 cells were incubated for 24 hours with or without the presence of CD40L transfected fibroblasts. After the stimulation, the DLBCL cells were harvested and stained for Mcl-1, Bcl-2 and Bcl-xL, and then assessed by using flow cytometry. Statistical analysis was performed on five independent experiments' Mean Fluorescence Intensity (MFI) values, between unstimulated and CD40L stimulated SUDHL8 cells, (*P<0.05, **P<0.01 paired T-test). (B) SUDHL8 cells were incubated for 24 hours with or without the presence of fibroblasts. After the stimulation, the DLBCL cells were harvested and stained for Mcl-1, Bcl-2 and Bcl-xL, and then assessed by using flow cytometry. Statistical analysis was performed on three independent experiments Mean Fluorescence Intensity (MFI) values, between the monocultured and co-cultured SUDHL8 cells, (*P<0.05, paired T-test).

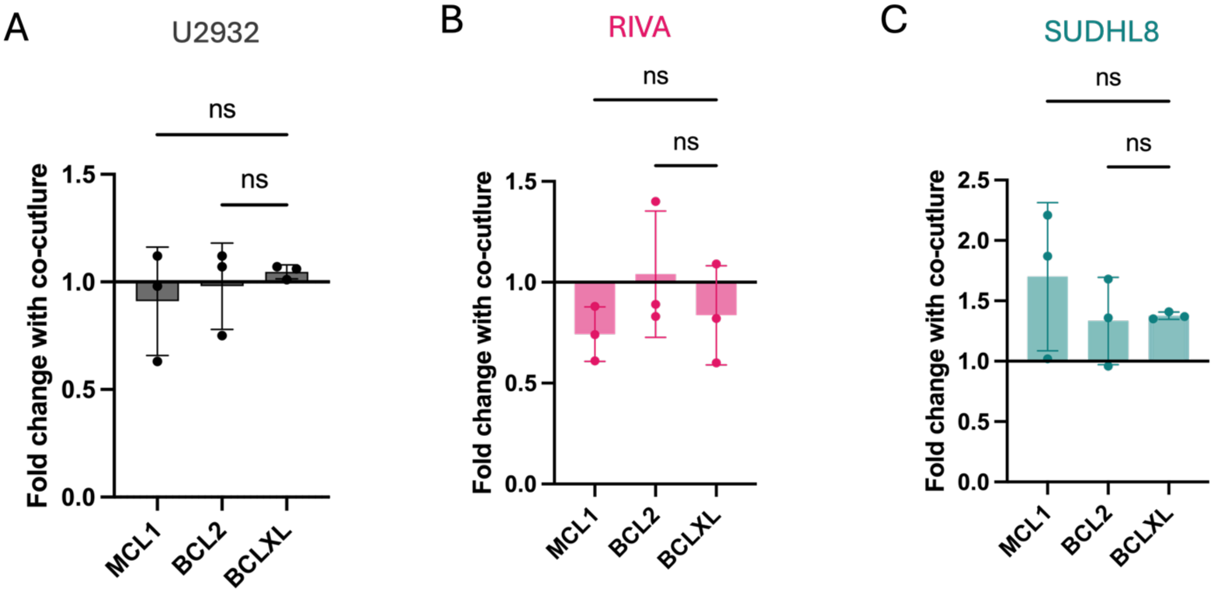

Supplemental Figure 4. BCL2-family levels in Diffuse Large B Cell Lymphoma (DLBCL) cell lines, after co-culture with NIH3T3 fibroblasts. (A) MCL1, BCL2, and BCLXL levels, shown as fold changes of 24-hour stimulation with NIH3T3 cells to unstimulated control in U2932 cells with error bars representing the mean ± standard deviation of three independent experiments *(one-way ANOVA with Tukey's comparisons test)*. (B) MCL1, BCL2, and BCLXL levels, shown as fold changes of 24-hour stimulation with NIH3T3 cells to unstimulated control in RIVA cells with error bars representing the mean ± standard deviation of three independent experiments *(one-way ANOVA with Tukey's comparisons test)*. (C) MCL1, BCL2, and BCLXL levels, shown as fold changes of 24-hour stimulation with NIH3T3 cells to unstimulated control in SUDHL8 cells with error bars representing the mean ± standard deviation of three independent experiments *(one-way ANOVA with Tukey's comparisons test)*.

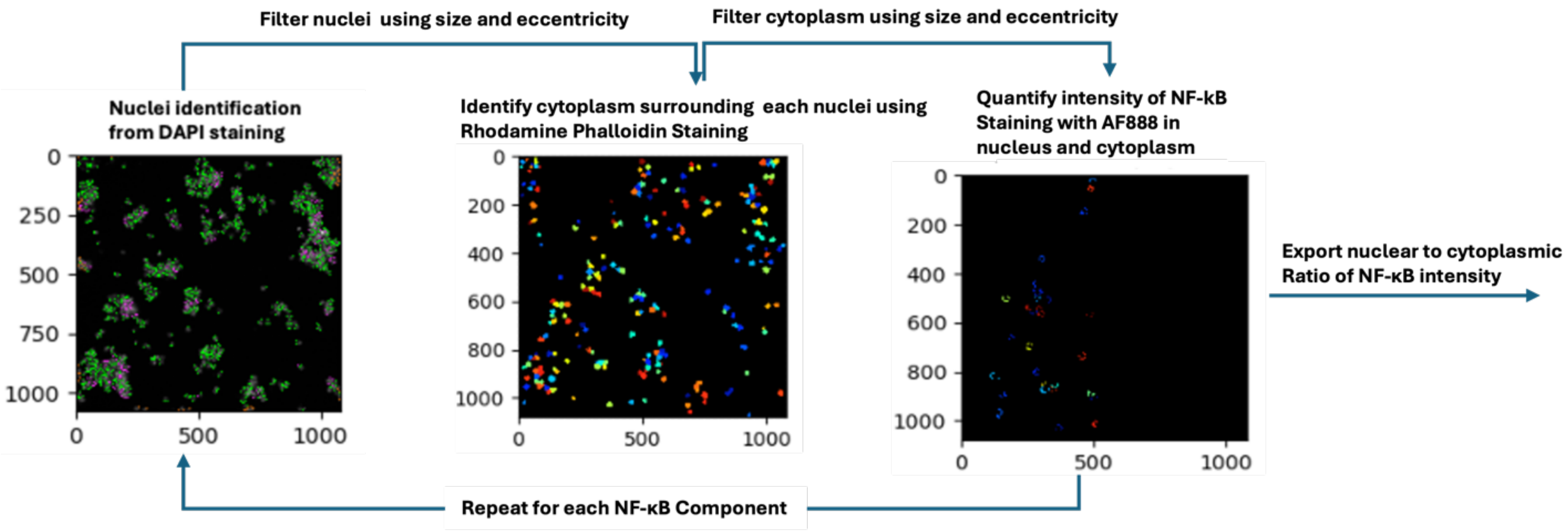

Supplemental Figure 5. Automated CellProfiler Pipeline for Nuclear:Cytoplasmic NF-κB analysis. The schematic diagram outlines the analysis performed from the original image. Cells were stained with DAPI to detect the nucleus with the Blue channel, Rhodamine Phalloidin detected with the Red channel to identify the cytoplasm, and AF488 detected from the Green channel to identify NF-κB (RelA, RelB or cRel). Nuclei and cytoplasm were segmented based on size and eccentricity, prior to overlay with AF488 for the quantification of nuclear to cytoplasmic NF-κB ratio for each individual cell with defined nucleus and cytoplasm. Images were acquired on 20x air setting of *OperettaCLS*.

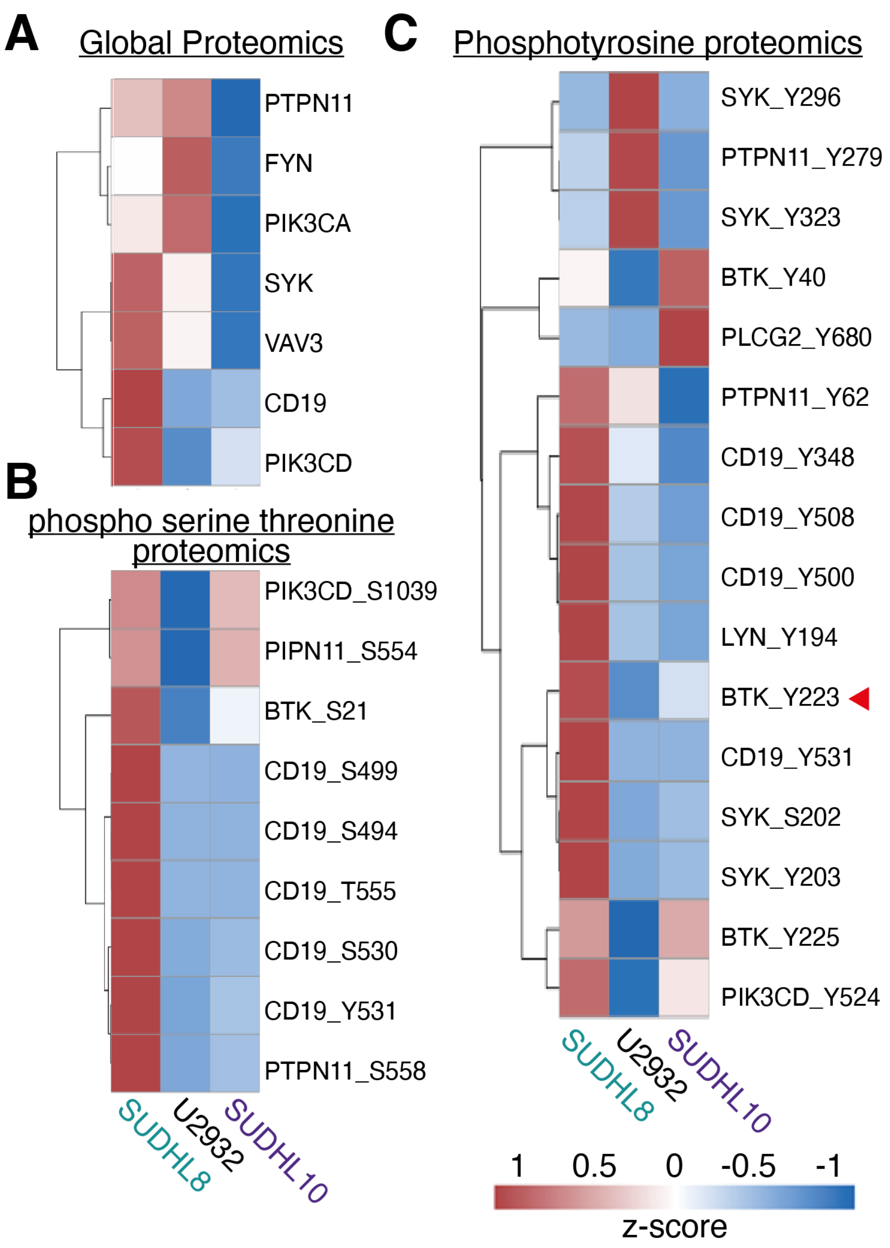

Supplemental Figure 6. Heatmap of B Cell Receptor (BCR) Signaling in SUDHL8, U2932, and SUDHL10 Cell Lines. (A) Global proteomics identified 7 BCR-associated proteins with higher expression in SUDHL8 compared to U2932 and SUDHL10. (B) Phospho-serine/threonine proteomics identified 9 BCR-related phosphorylation sites with elevated expression in SUDHL8. (C) Phosphotyrosine proteomics identified 16 BCR-related phosphorylation sites, including BTK_Y223, CD19_Y348, and SYK_Y203, showing higher expression in SUDHL8 compared to the other cell lines.

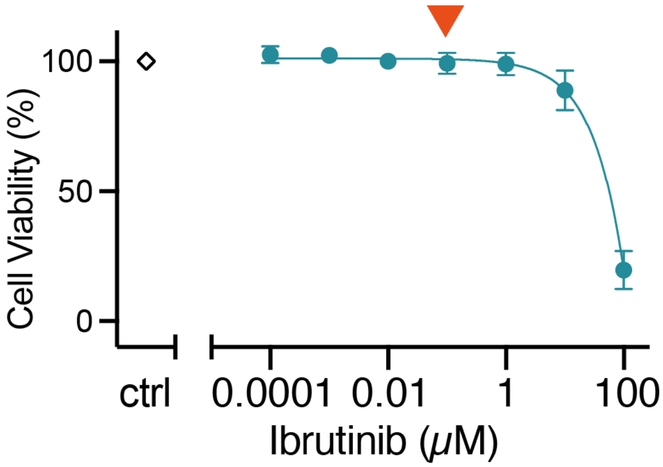

Supplemental Figure 7. Cell viability of SUDHL8 cells in response to 0.0001-100μM of Ibrutinib 24-hour treatment with BTK-inhibitor ibrutinib. Error bars representing the mean ± standard deviation of three independent experiments, normalized to the untreated control. The dose used for resensitization in Figure 6H is indicated with an arrow.

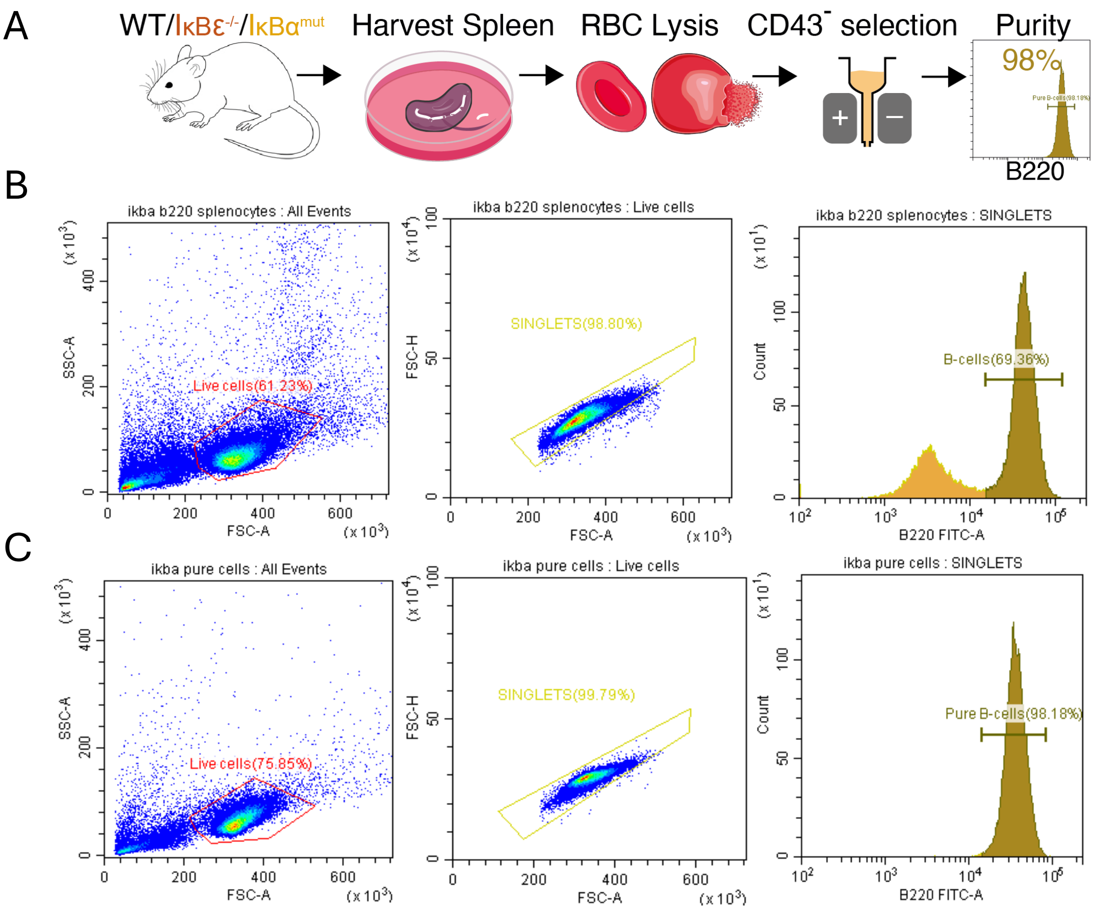

Supplemental Figure 8. B cell isolation and purification from primary splenocytes**.** (A) Schematic of B cell isolation and purification from mouse spleens. (B) Flow cytometry gating example on B220$^+$ B cells from homogenized mouse splenocytes. (C) B220$^+$ gating on mouse B cells, post CD43$^-$ magnetic column selection to assess B cell purity percentages.

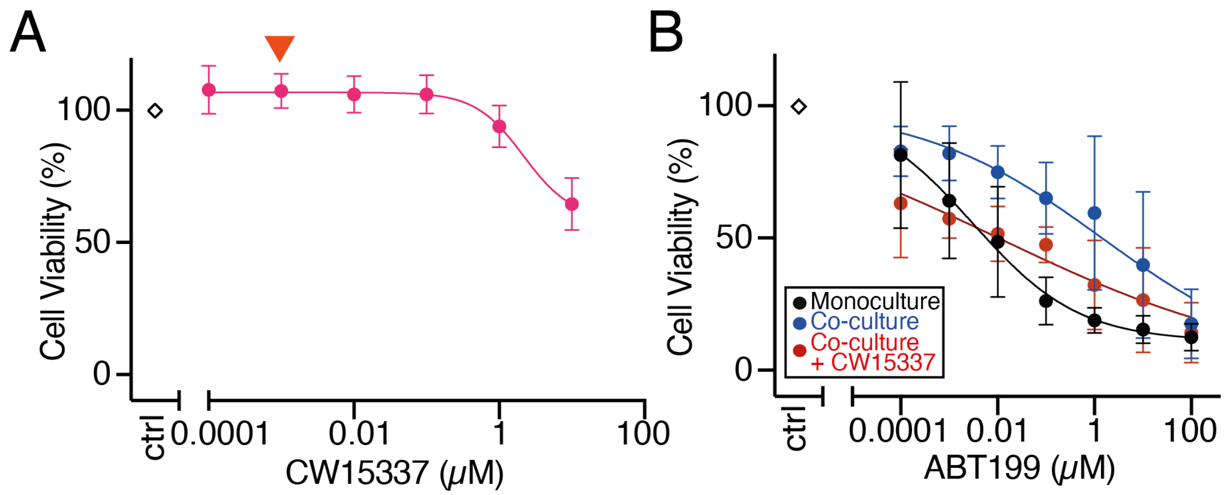

Supplemental Figure 9. Low doses of NIK inhibitor CW15337 can overcome tumor microenvironment (TME) resistance in RIVA cells. (A) Cell viability of RIVA cells in response to 0.0001-100μM 24-hour treatment with the NIK inhibitor CW15337. Error bars representing the mean ± standard deviation of three independent experiments, normalized to the untreated control. The dose used for resensitization in panel B is indicated with an arrow. (B) Cell viability of RIVA in response to 0.0001-100μM of the BCL2 inhibitor ABT199 post a 24-hour treatment in monoculture, post a 24-hour hCD40L-3T3 co-culture or post a 24-hour hCD40L-3T3 co-culture with the addition of 0.001μM of the NIK inhibitor CW15337. Error bars representing the mean ± standard deviation of three independent experiments, normalized to the untreated control.

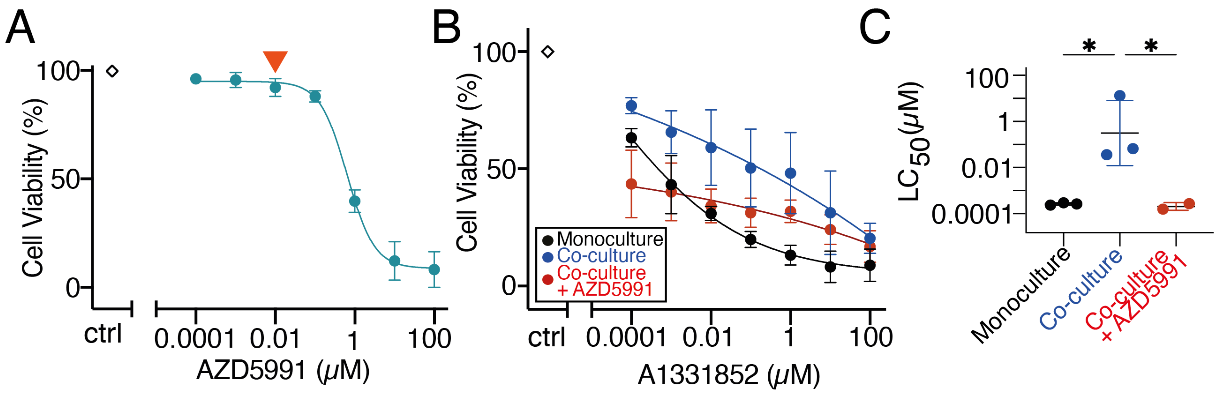

Supplemental Figure 10. Targeting MCL1 can overcome tumor microenvironment (TME) resistance in SUHL8 cells. (A) Cell viability of SUDHL8 cells in response to 0.0001-100μM 24-hour treatment with the MCL1 inhibitor AZD5991. Error bars representing the mean ± standard deviation of three independent experiments, normalized to the untreated control. The dose used for resensitization in panel B is indicated with an arrow. (B) Cell viability of SUDHL8 in response to 0.0001-100μM of the BCLXL inhibitor A1331852 post a 24-hour treatment in monoculture, post a 24-hour hCD40L-3T3 co-culture or post a 24-hour hCD40L-3T3 co-culture with the addition of 0.01μM of the MCL1 inhibitor AZD5991. Error bars representing the mean ± standard deviation of three independent experiments, normalized to the untreated control. (C) LC_{50} values for each condition is shown with error bars representing the mean ± standard deviation of three independent experiments, normalized to the untreated control (*$P<0.05$ one-way ANOVA with Tukey's comparisons test).

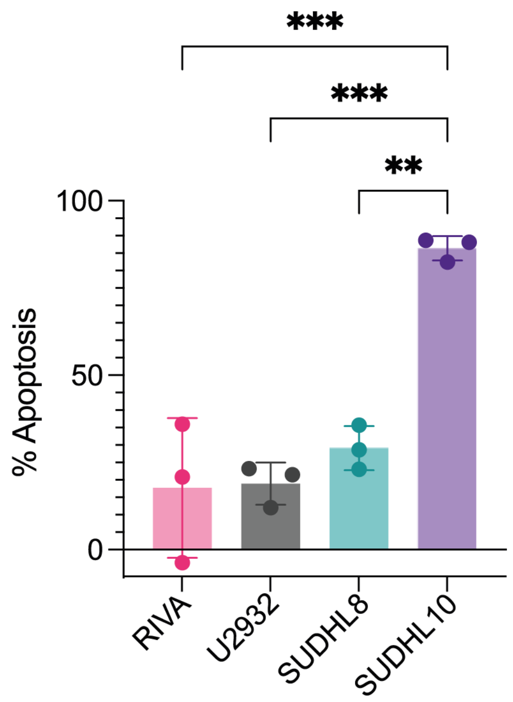

Supplemental Figure 11. Percentage of apoptotic cells in response to 50μM of Amgen16 in the indicated DLBCL cell lines post a 24-hour treatment, error bars representing the mean ± standard deviation of three independent experiments, normalized to the untreated control. (**P<0.01, ***P<0.001; one-way Anova with Tukey's multiple comparisons). This is a subset and re-plotting of the data displayed in Figure 2D.

Supplemental modelling description.

Computational simulations of SUDHL8 and RIVA cells (Figure 6A and B) were performed using a published model of NF-kB/TLR/BCR signalling (Jayawant et al. Frontiers in Immunology, 2023). This model can be run using the code provided here: https://github.com/SiFTW/VareliEtAl/tree/main/modelling.

Each model simulations have three input curves representing toll like receptor (TLR), B cell receptor (BCR), and NF-κB-inducing kinase (NIK) activity. The only difference between the two simulations it he level of BCR activity, which for the RIVA cells is 0 and for SUDHL8 cells is 0.005. All other parameters for both cell line simulations are identical. In each cell line's simulation BCR activity is maintained at its defined level through out and does not change over time.

Each simulation has two phases. One "steady state" phase in which all input curves are fixed and do not change over time, and a "time course" phase where NIK activity increases over time to represent the effect of co-culturing the cells with CD40L-expressing cells. At the end of the steady state phase we calculated how much RelA:p50 and cRel:p50 is bound to each IκB in the simulation with normal BCR activity (U2932) and high BCR activity (SUDHL8). This data is shown in Figure 6A. We then run the time course phase in which NIK activity increases. The NIK activity profile in both simulated cell lines is the same, the only difference between them remains the higher BCR activity in the SUDHL8 cell line. This effect of this increasing NIK activity on nuclear RelA:p50, RelB:p52, and cRel:p52 is shown in Figure 6B.

In Figure 6B we see an increase in nuclear RelB:p52 because increasing NIK activity processes p100 into p52 to enale the formation of RelB:p52. Only in SUDHL8 cells do we also see an increased nuclear translocation cRel:p50. This is because NIK activity also degrades IκBδ. In Figure 6A we saw that IκBδ was strongly inhibiting cRel:p50 only in the SUDHL8 cells, and this is why we only see induction of cRel:p50 in response to NIK increases in this cell line.

Supplemental Table 1: Antibodies used for flow cytometry, immunofluorescent microscopy (IF) and western blotting

**Antibody**	**Fluorophore**	**Clone**	**Company**	**Catalog Number**	**Dillution µl:µl**
CD20	BV 711	2H7	BioLegend	302342	2.5:100
B220	FITC	RA3-6B2	BioLegend	103206	2.5:100
BCL2	BV 421	100	BioLegend	658709	1.25:100
MCL1	PE	D2W9E	Cell Signalling	65617S	1:100
BCLXL	APC	54H6	Cell Signalling	12099S	2:100
RelA	APC	14G10A21	BioLegend	654006	5:100
RelB	488	pAb	Proteintech	CL488-25027	1:200
cRel	FITC	1RELAH5	Fisher	12-6111-80	2.5:100
RelA (IF)	-	D14E12	Cell Signalling	8242S	1:400
RelB (IF)	-	pAb	Proteintech	25027-1-AP	1:200
cRel (IF)	-	JM72-93	Fisher	MA5-32745	1:200
Nuclei Stain	DAPI	-	Fisher	10116287	1:500
Rhodamine Phalloidin	TRITC	-	ThermoFisher	R415 10063052	1:400
AF 488	AF488	pAb	Invitrogen	10729174	1:500
p100/p52	-	W16082A	BioLegend	640945	5:1000
AF 680	AF680	pAb	Fisher	A-21096	1:2000
Annexin V	FITC	-	BioLegend	640945	2.5:100
