# Supplementary figures and images for: Systems biology-enabled targeting of NF-κΒ and BCL2 overcomes microenvironment-mediated BH3-mimetic resistance in DLBCL
Source: Cell Death Dis. 2025 Aug 16;16(1):620. doi: 10.1038/s41419-025-07942-0 (PMC12357900; doi:10.1038/s41419-025-07942-0)

Uncropped WB p100/p52
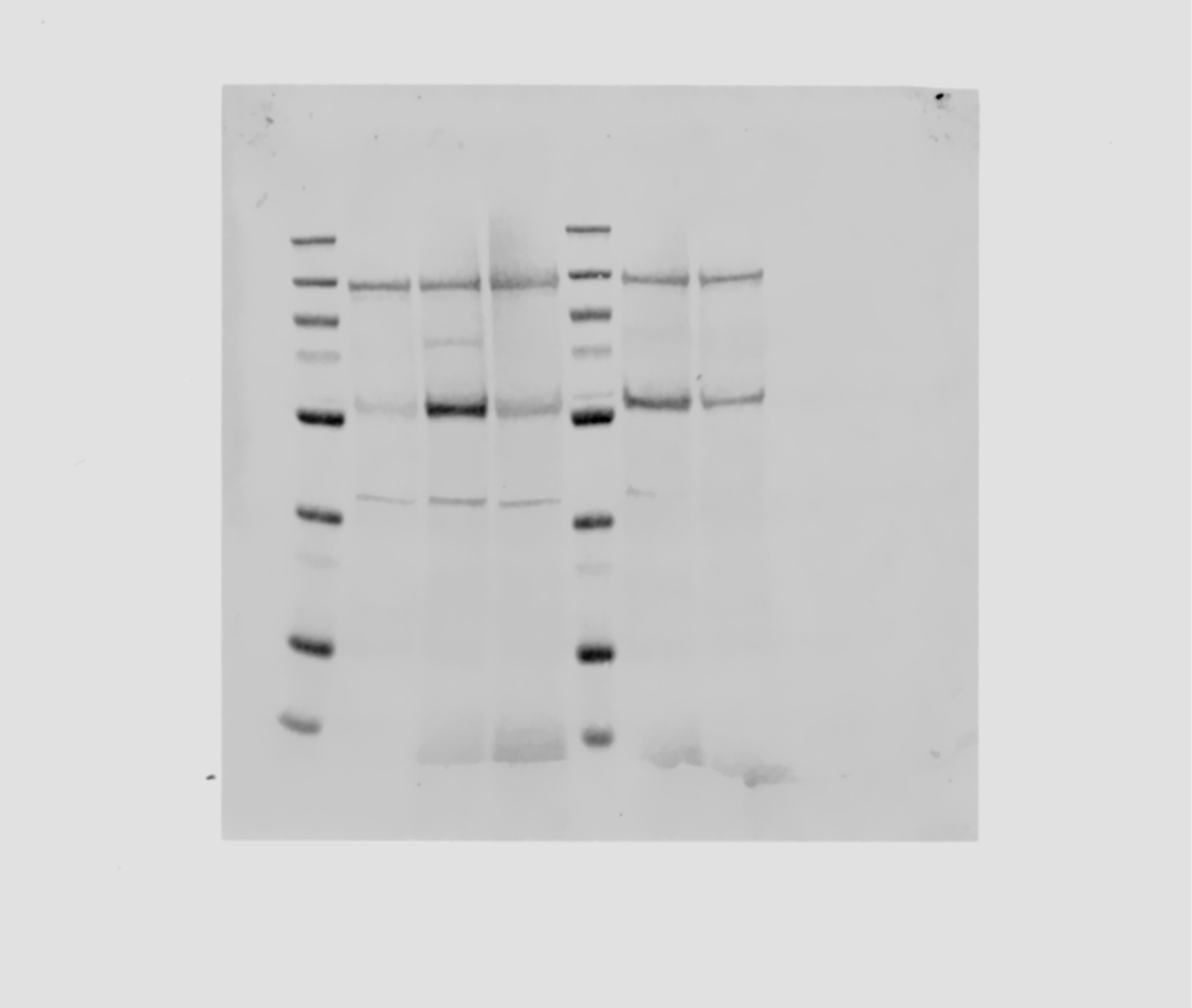


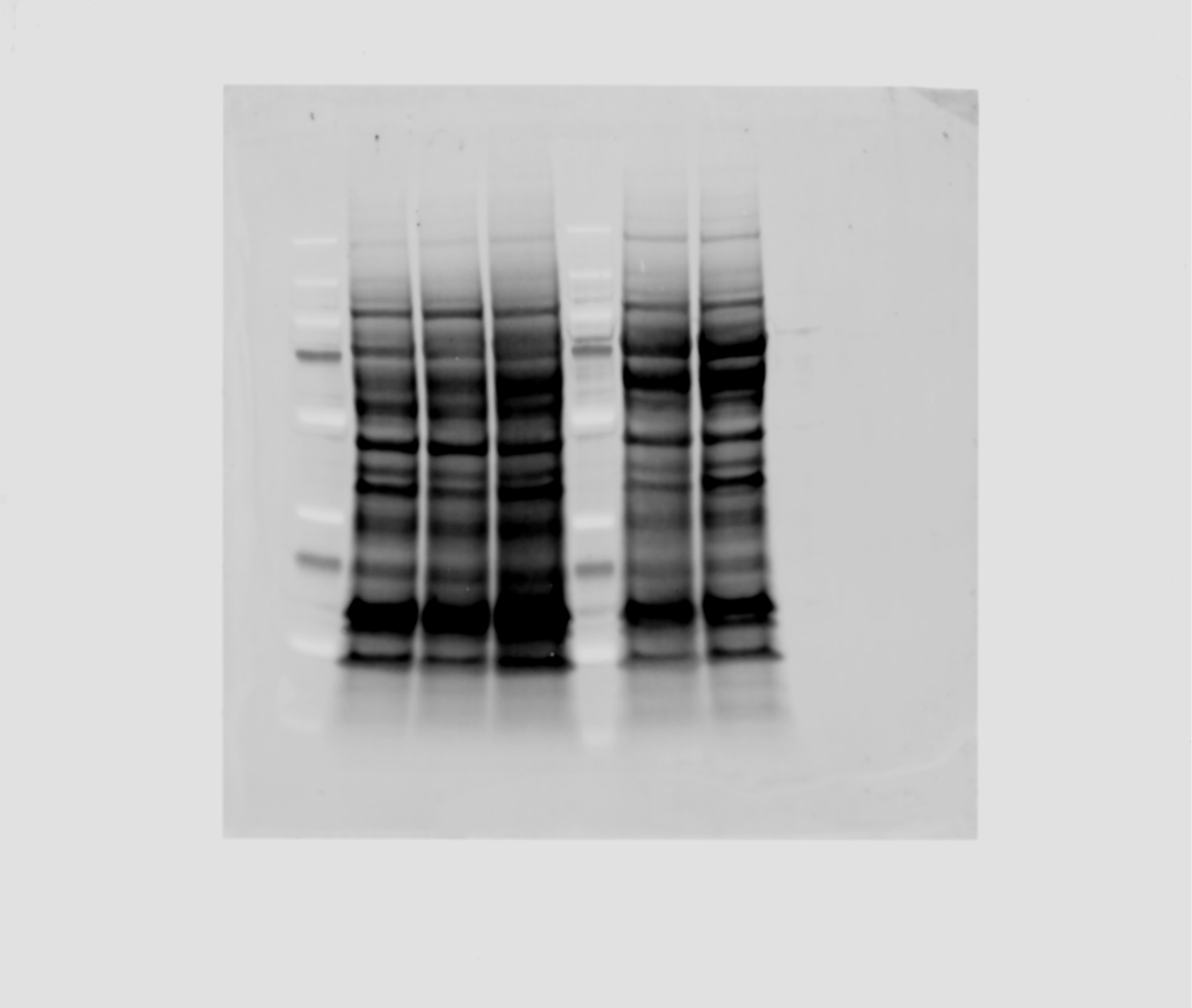
Uncropped WB (total protein)

Supplement: Supplementary file 2 — original data [file 41419_2025_7942_MOESM2_ESM.docx]
